# Effect of preoperative oral antibiotics in combination with mechanical bowel preparation on inflammatory response and short‐term outcomes following left‐sided colonic and rectal resections

**DOI:** 10.1002/bjs5.50283

**Published:** 2020-04-11

**Authors:** O. Hartrick, S. Pengelly, R. Bethune

**Affiliations:** ^1^ Colorectal Surgery, Royal Devon and Exeter NHS Foundation Trust Barrack Road Exeter EX2 5DW UK

Golder *et al*.'s propensity‐matched retrospective observational study found the addition of preoperative oral antibiotics and mechanical bowel preparation to prophylactic intravenous antibiotics was associated with reduced postoperative complications and severity of systemic inflammatory response^1^. We believe several statistical issues may have led to the treatment effect being overstated.

First, more patients in the control group had malignant disease; this can lead to a heightened inflammatory state. The *P* values that would have made this obvious were removed between *Table 1* and *Table 2*. Second, the historical nature of the control group, with unmeasured aspects of treatments likely to improve over time, will lead to improved outcomes in the treatment group. Taken together, these two effects could lead to false rejection of the null hypothesis.

Third, the postoperative Glasgow Prognostic Score (poGPS) is determined solely by albumin and C‐reactive protein (CRP) concentrations^2^. Determining statistical differences in poGPS at the same time as differences in CRP and albumin, at exactly the same cut‐off values, artificially inflates the number of hypotheses being tested. In addition, no Bonferroni correction for multiple hypothesis testing was undertaken for the 15 *P* values given in *Table 3*; again, this could lead to a type I error and is an example of *P*‐hacking.

We feel these points have led to Golder and colleagues overstating the likelihood of any treatment effect. Gut decontamination in colorectal surgery is an important issue that deserves a large high‐quality RCT.

## Disclosure

The authors declare no conflict of interest.
